# Supplementary material for: Vestibular and Auditory Hair Cell Regeneration Following Targeted Ablation of Hair Cells With Diphtheria Toxin in Zebrafish
Source: Front Cell Neurosci. 2021 Aug 19;15:721950. doi: 10.3389/fncel.2021.721950 (PMC8416761; doi:10.3389/fncel.2021.721950)
Supplement: Supplementary file 1 [file Data_Sheet_1.PDF]

Supplementary Material

Supplementary Data

Supplementary Video 1. DT induced hair cell death affects adult swimming behaviors. Injected wild-type fish (left panel) and Tg(*myo6b*:hDTR) with 10 ng of DT 3 days-post injection. Tg(*myo6b*:hDTR) display summersaulting and lateral looping when water is agitated.

Supplementary Figures

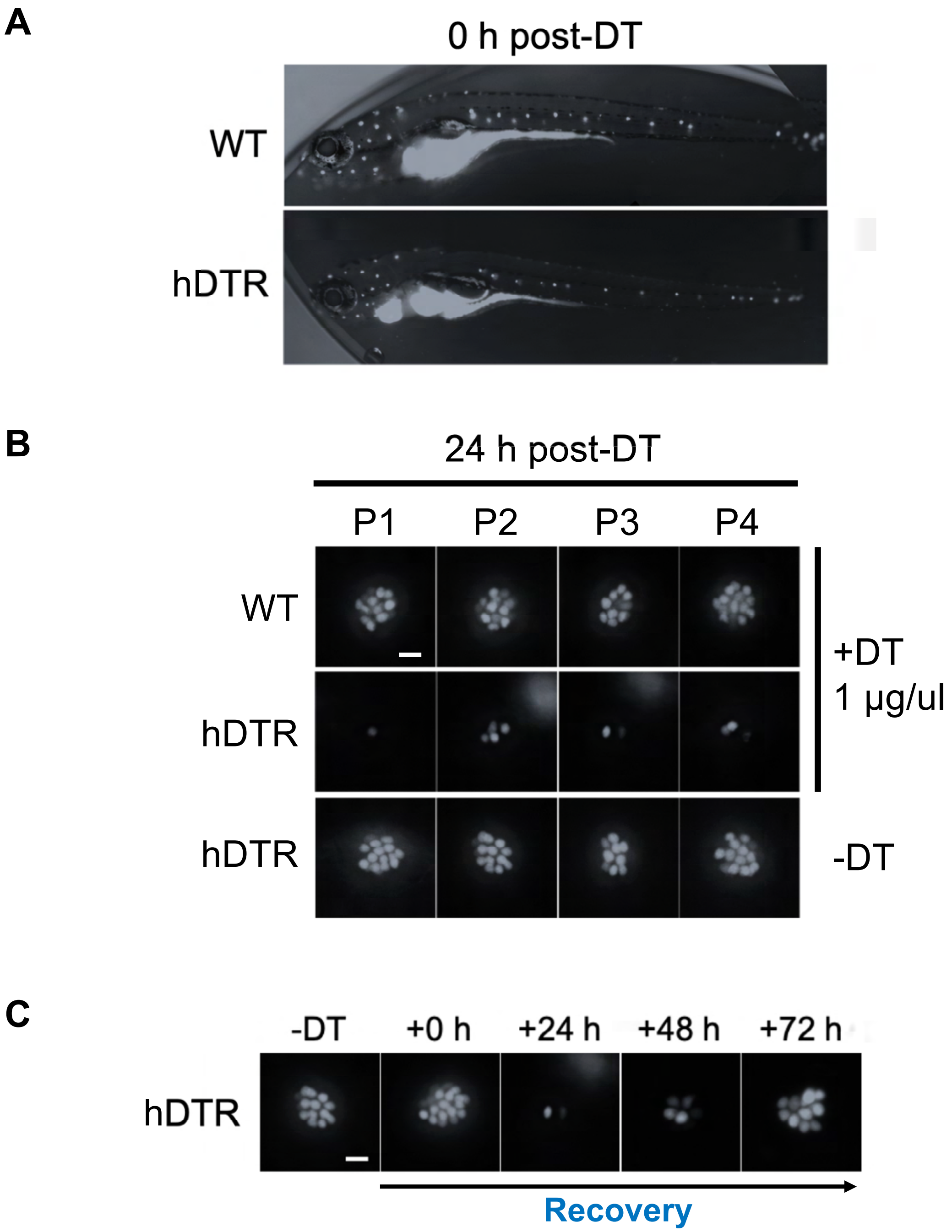

Supplementary Figure 1. Tg(*myo6b*:hDTR) larval zebrafish show hair cell loss and regeneration in lateral line neuromasts after *in vivo* DT treatment. Larvae were exposed to 1 µg/mL of DT for 6 h. Neuromast viability was monitored daily by YO-PRO-1 labelling 0 h to 72 h post-DT incubation. The 1 µg/mL concentration and the 6 h duration of DT exposure was selected based on dose-dependent work in Figure 2. **(A)** Wild-type (WT) controls and Tg(*myo6b*:hDTR) larvae (hDTR) 0 h post-DT. **(B)** Neuromasts 24 h post-DT in WT and Tg(*myo6b*:hDTR) larvae. **(C)** Regeneration time-course showing P3 neuromast of Tg(*myo6b*:hDTR) larvae treated with DT. Scale bar 100 µm.

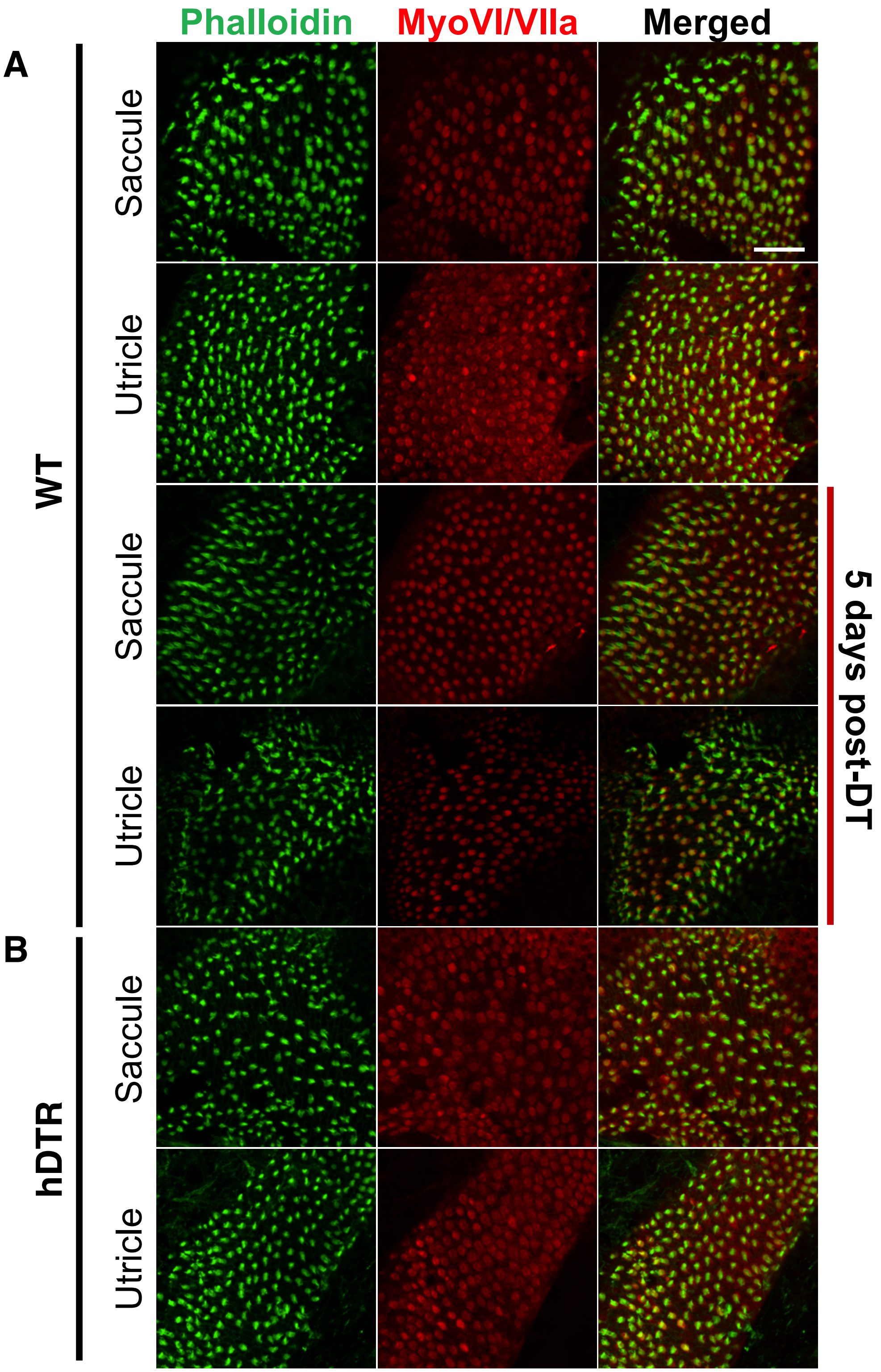

Supplementary Figure 2. Tg(*myo6b*:hDTR) sensory epithelia. (A) Untreated and treated saccule and utricle from wild-type fish (WT). Saccule and utricle were isolated from treated wild-type fish 5 days post-DT. **(B)** Untreated saccule and utricle from Tg(*myo6b*:hDTR) fish (hDTR). Close examination of hair cells with phalloidin (green channel) and hair cell bodies with anti-myosin VI/VIIa (red channel). Scale bar 20 μm.
